# Diagnosing migraine in children and adolescence using ID migraine: results of an Italian multicenter validation

**DOI:** 10.1007/s10072-025-08076-z

**Published:** 2025-03-27

**Authors:** Ilaria Frattale, Vittorio Sciruicchio, Daniela D’Agnano, Vincenzo Raieli, Salvatore Lo Cascio, Giuseppe Santangelo, Edvige Correnti, Fabiana Ursitti, Giorgia Sforza, Gabriele Monte, Luigi Mazzone, Massimiliano Valeriani, Laura Papetti

**Affiliations:** 1 Child Neurology and Psychiatry Unit, Department of Wellbeing of Mental and Neurological, Dental and Sensory Organ Health, Policlinico Tor Vergata Foundation Hospital, Rome, Italy; 2 Children Epilepsy and EEG Center, PO San Paolo, ASL Bari, Italy; 3 Child Neuropsychiatry Department, ISMEP-ARNAS Civico Palermo, Via Dei Benedettini 1, 90100 Palermo, Italy; 4 Child Neuropsychiatry Unit Department, Pro.M.I.S.E. “G D’Alessandro”, University of Palermo, Palermo, Italy; 5 Developmental Neurology, Bambino Gesù Children Hospital, IRCCS, Rome, Italy; 6 System Medicine Department, Tor Vergata University of Rome, Rome, Italy; 7 Center for Sensory-Motor Interaction, Aalborg University, Aalborg, Denmark

**Keywords:** Migraine, ID Migraine, Children, Pediatric, Screening tool

## Abstract

**Background:**

Since migraine is the most frequent neurological condition, an early diagnosis is important to limit the impact of the disease on the quality of life. Although migraine diagnosis is based on the International Classification of Headache Disorders 3rd edition (ICHD3) criteria, other briefer questionnaires have been developed, especially for screening purpose. While the three-item ID Migraine has proved useful for migraine diagnosis in adulthood, no validated tools are available for children and adolescents. The aim of this study is to validate ID Migraine also in pediatric patients.

**Results:**

The Italian ID Migraine for adulthood was completed by 289 pediatric patients (mean age 12.14 ± 3.15, range 6–17) who attended three third-level pediatric headache centers. Clinical and neurological examinations were performed, and the final diagnosis was reached according to the ICHD3 criteria. The migraine group consisted of 230 patients, and the control group consisted of 59 patients who received headache diagnoses different from migraine. We considered the ID migraine positive whether 2 out of 3 responses were 'yes'. ID migraine for diagnosis of pediatric migraine showed a sensitivity of 0.86 (86%), a specificity of 0.95 (95%), a positive predictive value (PPV) of 0.98 (98%) and a negative predictive value (NPV) 0.64 (64%).

**Conclusion:**

ID Migraine can be considered a valid tool for migraine diagnosis also in pediatric age, starting from the age of 6 years.

## Background

Migraine is the most frequent neurological condition afflicting both adult and pediatric age [1, 2], with the highest rate of burden of disease, linked to medical costs, instrumental tests, school and work absences [3].

The diagnosis is clinical, according to the International Classification of Headache Disorders version 3 criteria [4].

An easily and quickly administrable questionnaire makes migraine diagnosis feasible also in settings other than a neurology unit, such as a pediatric or emergency department, where the right diagnosis favors clinical framework and instrumental diagnostic process.

The three-item ID Migraine is a screening questionnaire consisting of 3 questions, administrable between the ages of 18 and 65 [5], whose purpose is to diagnose migraine on the base of the main features associated with the attack, such as nausea or vomiting, photophobia and disability.

ID Migraine has been validated in many countries and many studies have used ID Migraine as diagnostic tool for migraine in adulthood [6–20]. Fewer studies are available on its applicability for diagnosing migraine in childhood [21–23].

The applicability of the ICHD3 criteria for migraine in childhood is debated due to the quality pain, which is rarely described throbbing as in adulthood [24], the duration of the attacks, often shorter than 2 h [4, 25], and the possible inability to explain associated symptoms, such as photo and phonophobia [24]. Considering these features, the expectation is that the ID migraine could be valid for the adolescence, when the characteristics of the migraine attacks are more similar to those of the adulthood, but not for younger children, due to migraine features which do not perfectly match the ICHD3 criteria [24, 25].

An Italian version of ID Migraine is available for adulthood [26]. For pediatric age, to date, no validated diagnostic tools are available. Two prior studies have tried to demonstrate the applicability and validity of ID Migraine in childhood with conflicting results [23, 27].

The aim of the present study is to demonstrate the validity of ID Migraine questionnaire, commonly used in adult patients, also in children and adolescents.

## Methods

Patients attending the Headache Centers of the Bambino Gesù Children’s Hospital in Rome, San Paolo Hospital in Bari and Civico Hospital in Palermo (Italy), in the period between January 2022 and February 2024 were included in the study.

The inclusion criteria were: 1) aged 6–17 patients with migraine with and without aura and other primary headache diagnosis according to ICHD3 criteria; 2) parents’ consent to answer the questionnaire.

The exclusion criteria were: 1) age over 17 and less than 6; 2) tension type headache diagnosis; 3) headaches attributed to secondary causes; 4) intellectual disability.

Part of the population was composed of individuals included in a previous preliminary validation study conducted only in the Rome center [28].

The original ID-Migraine, validated by Lipton et al. [5] for adulthood, consists of 3 questions, which investigate the presence of the main features associated to the migraine attack, such as nausea or vomiting, photophobia and disability [considered as the inability to carry out normal daily activities during the attack]. The dichotomous responses are coded as “no” (meaning never or rarely) and “yes” for each question. The questionnaire is considered as supporting migraine diagnosis if 2 out of 3 answers are “yes”.

**Fig. 1:**
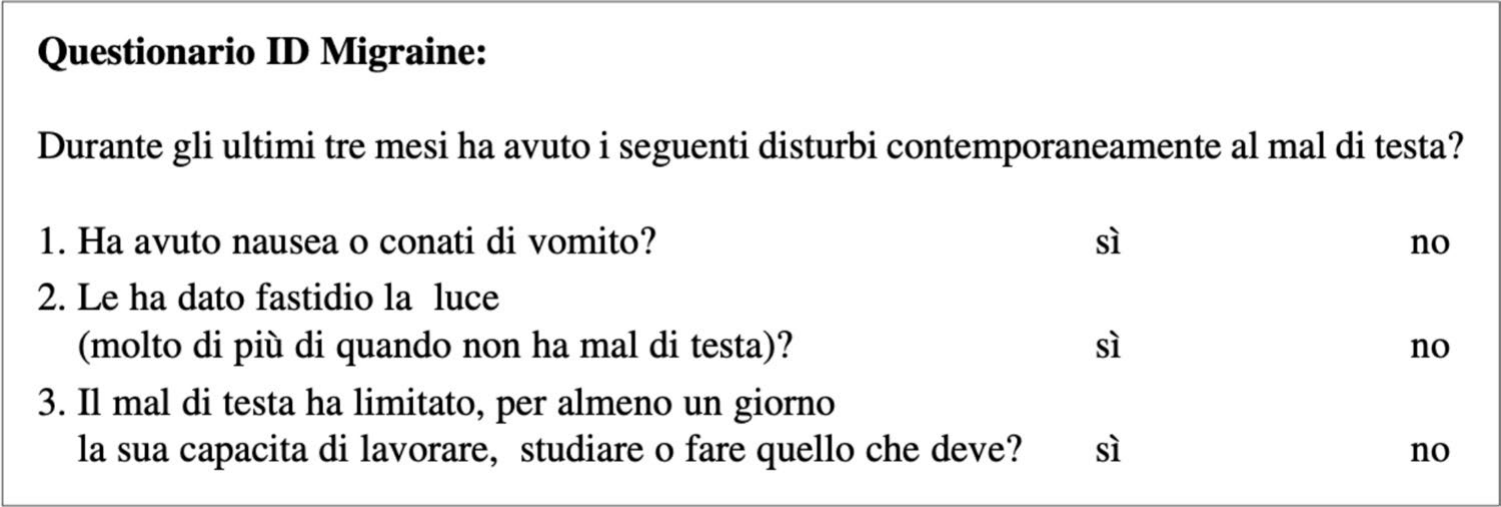
Italian ID Migraine validated by Brighina et el. translated from the original version [5]: During the last 3 months, did you have the following disturbances with your headaches? 1. Did you have nausea or retching? Yes / No 2. Did light bother you [more than when you did not have headache]? Yes / No 3. Did your headache limit your ability to work, study, or do what you needed to do? Yes / No

In the present study, we used the questionnaire validated in Italian for adult migraineurs by Brighina et al. [26] (Fig. 1), who gave consent to its use.

After ID Migraine administration, patients underwent clinical evaluation, including neurological and general exam, and fundus oculi. Two neurologists, specialized in headache medicine, blinded to the previous evaluation, confirmed the headache diagnosis based on the ICHD3 criteria.

According to the ICHD3 diagnosis, patients were assigned to two groups: “migraine” and “other primary headache disorders”. Patients with tension-type headache according to ICHD 3 were excluded from the analysis. The reason is that tension-type headache diagnosis in pediatric age is underrated with relevant troubles in differential diagnosis with migraine [29, 30]. In childhood, tension-type headache and migraine are considered a continuum of the same spectrum, with characteristics attributable to both diagnoses in the same patient [25, 31].

The category “other primary headache disorders” included patients with diagnosis of SUNA/SUNCT [Short-lasting unilateral neuralgiform headache with cranial autonomic symptoms/ short-lasting unilateral neuralgiform headache with conjunctival injection and tearing], cluster headache and primary stabbing headache.

The diagnosis obtained by the ICHD3 criteria was matched with the answers provided to the ID-migraine and included in a unique database.

The population was stratified into two groups on the base of a cut-off settled at 11 years: under or equal 11 (children) and over 11 (adolescents).

Descriptive statistics were performed and results are presented as sensitivity, specificity and negative (NPV) and positive predictive (PPV) values. These were considered reliable for values > 80%. The Wilcoxon Rank-Sum test and the Chi-Square test were used for the analysis of categorical variables.

Statistical analysis was conducted using SPPS version 22.0.

The study was approved by the Ethical Committee of Bambino Gesù Children’s Hospital. All patients enrolled and their parents provided consent for the publication of the results.

## Results

**Fig. 2:**
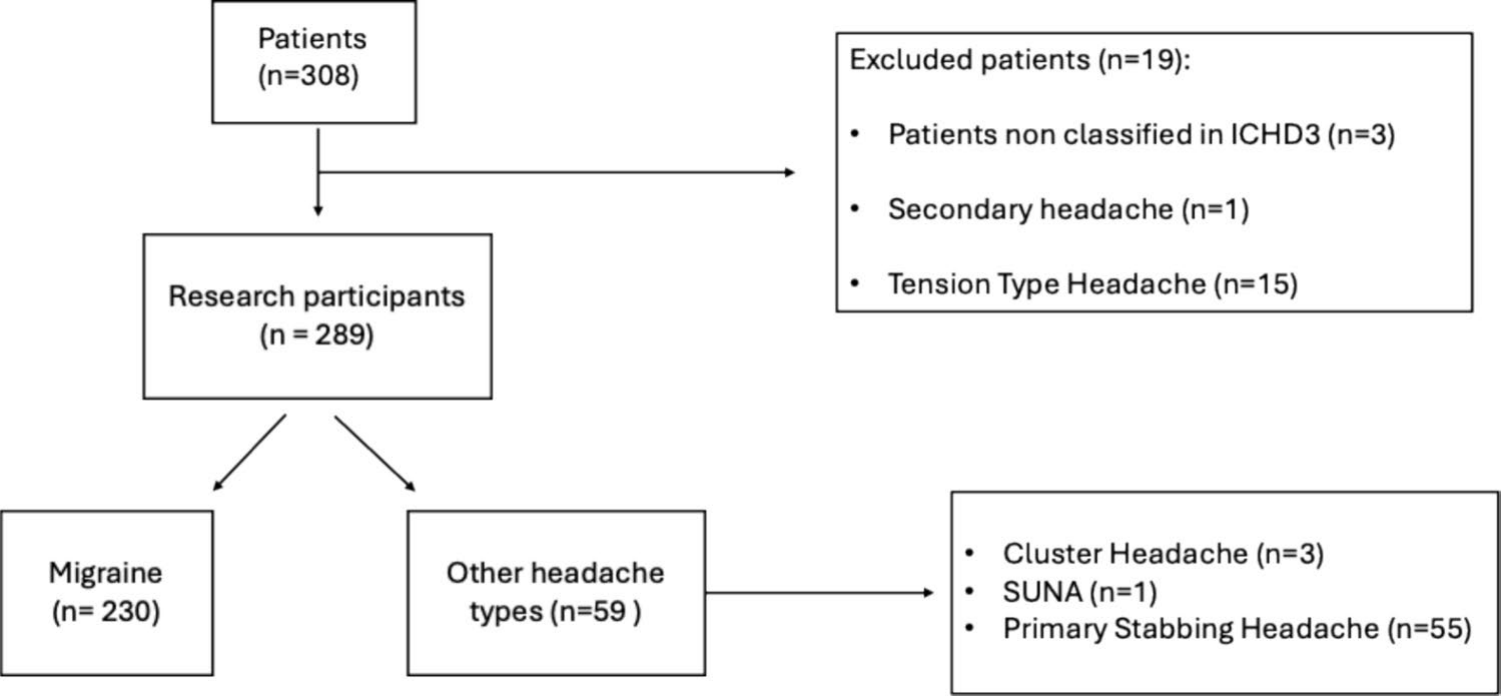
Study population flowchart

A total of 289 patients (169 girls, 120 boys) who gave consent to the questionnaire administration were included. The mean age of the studied population was 12.14 years (median age 12.16 years, SD ± 3.15, range 6–17 years). The study population flowchart is reported in Fig. 2.

Migraine cohort consisted of 230 subjects, of whom 9.1% with migraine with aura; there were 133 females (58%) and 97 males (42%); the mean age of the migraine group was 12.35 years (median age 12.42 years, SD ± 2.97, range 6–17 years).

The control group included 59 patients with any headache different from migraine and tension type headache, including primary stabbing headache (93%) and TACs (7%). Fifty-nine patients were considered as controls (36 females—61%, and 23 males—39%). Their mean age was 11.34 years (median age 11 years, SD ± 3.71, range 6–17 years).

Demographic features of the total included population are reported in Table 1.

**Table 1:** Demographic features of 289 included patients

Total Patients:	289
Mean age	12.14 ± 3.15
Female: male [%]	169:120 (59%—41%)
Migraine Group	*n* = 230
Migraine without aura	*n* = 209 (91%)
Migraine with aura	*n* = 21 (9%)
Female:Male [%]	133: 97 (58%—42%)
Mean age ± SD	12.35 ± 2.97
≤ 11 years old [%]	65 (28%)
> 11 years old [%]	165 (72%)
Control Group	*n* = 59
Primary Stabbing Headache	*n* = 55 (93%)
TACs	*n* = 4 (7%)
Cluster Headache	*n* = 3
SUNA	*n* = 1
Female:Male [%] Mean age ± SD ≤ 11 years old [%] > 11 years old [%]	36:23 (61%- 39%) 11.34 ± 3.71 26 (44%) 33 (56%)

TACs: Trigeminal Autonomic Cephalalgias; SUNA Short-lasting unilateral neuralgiform headache with cranial autonomic symptoms

Considering the individual ID-Migraine features, nausea/vomiting was reported in 144/230 patients with migraine (63%) versus 7/59 patients in the control group (12%), photophobia in 167/230 migraineurs (73%) versus 11/59 of the control group (19%), and disability was reported by 205/230 migraine patients (89%) versus 13/59 control patients (22%). Two positive responses were reported in 198/230 (86%) migraineurs versus 3/59 (5%) patients of the control group.

**Fig. 3:**
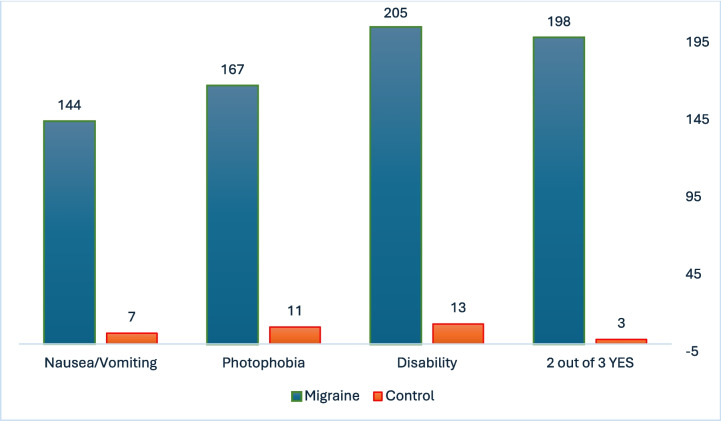
ID Migraine responses in the migraine and control group

The provided ID-Migraine responses are shown in Fig. 3.

Specificity, sensitivity and negative (NPV) and positive (PPV) predictive values ​​of the responses provided to the ID Migraine items are reported in Table 2.

**Table 2:** Specificity, sensitivity and negative (NPV) and positive (PPV) predictive values ​​of the responses provided to the ID Migraine

	Sensitivity (%)	Specificity (%)	PPV (%)	NPV (%)
2 “YES” responses	0.86 (86%)	0.95 (95%)	0.98 (98%)	0.64 (64%)
Nausea/Vomiting	0.62 (62%)	0.88 (88%)	0.95 (95%)	0.38 (38%)
Photophobia	0.73 (73%)	0.81 (81%)	0.94 (94%)	0.44 (44%)
Disability	0.89 (89%)	0.76 (76%)	0.94 (94%)	0.64 (64%)

**Fig. 4:**
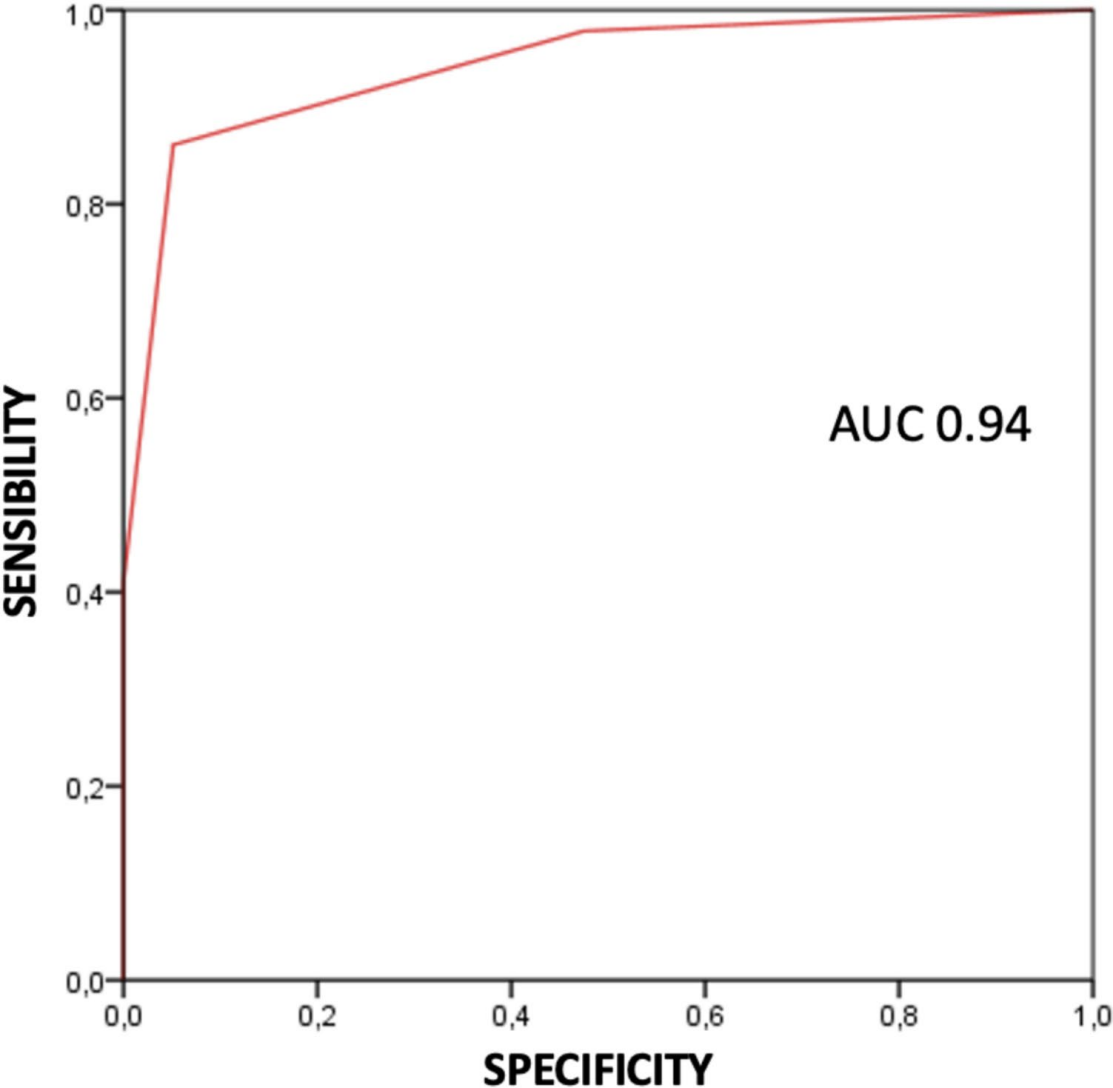
ROC value of the entire study population

Since the ID Migraine is commonly considered as suggesting migraine diagnosis when there are at least 2 “yes” responses, sensitivity of the questionnaire was 0.86 (86%), specificity 0.95 (95%), PPV 0.98 (98%), and NPV 0.64 (64%). Figure 4 shows the receiver operating characteristic (ROC) curve according to specificity and sensibility of the ID Migraine test (Fig. 4).

We also calculated the ID Migraine accuracy separately in children and adolescent patients and the results were similar between groups. In the children group, sensitivity was 0.87 (87%), specificity 0.92 (92%), PPV 0.97 (97%), and NPV 0.75(75%). In the adolescents’ group, sensitivity was 0.85 (85%), specificity 0.97 (97%), PPV 0.99 (99%), and NPV 0.57 (57%).

Considering the demographic features of the two subgroups, a total of 91 patients were included in the younger group, of which 48 were female (52.7%), and a mean age of 8.58 years (median age 9 years, SD ± 1.73, range 6–10.9 years). The elderly group consisted of 198 adolescents, with a female: male ratio of 120:78 (60.6%: 39.4%), and a mean age of 14.75 years (median age 14.3 years, SD ± 2.13 years, range 11–17 years).

## Discussion

The results of the present study show that ID Migraine is a valid diagnostic tool even in children and adolescents. In both in children and adolescents, the ID Migraine showed an excellent profile in terms of sensitivity (85% and 87%, respectively), specificity (97% and 92%, respectively) and PPV (99% and 97%, respectively).

The diagnosis of migraine in pediatric age is often challenging. The ICHD 3 criteria are designed primarily for adults and may not fully account for the peculiarities of pediatric migraine [24]. The unique ways in which children experience and express migraine symptoms, combined with the variability in presentation, require careful consideration. Children may not fully understand or communicate their symptoms, making them dependent on caregivers to interpret and report their experiences. This can lead to underreporting or misinterpretation of the severity and frequency of symptoms. Young children may have trouble describing their pain, aura, or associated symptoms [24, 32]. Children may not meet all the ICHD 3 criteria for migraine diagnosis [25, 29, 33–39] and a consensus of pediatric headache experts proposes some recommendations in order to make criteria more appropriate for children [24].

Pediatric migraine attacks tend to be shorter in duration than those in adults. Migraine episodes in children may last as short as 2 h, making it harder to fit them into traditional diagnostic criteria, which typically require headaches to last 2 h or longer [4, 25, 29, 40]. Unlike adults, children may not always experience typical migraine symptoms such as a unilateral throbbing headache. Bilateral and less severe pediatric pain can lead to underdiagnosis or misdiagnosis [34].

In childhood and adolescence, determining a definitive diagnosis of primary headache can be hindered also by the change in headache type. According to report, 8–32% of migraine sufferers transition to TTH, and 4–41% of TTH sufferers’ transition to migraine [41]. There are two possibilities to explain the observed changes of childhood headache patterns. First, the 'continuum severity theory' states that primary headache is a continuum between TTH and migraine. In this model, headache is labelled tension-type when the pain is mild, as common migraine when the pain is more severe, and as classic migraine when the pain is associated with neurological symptoms [42]. Secondly, it is possible for children to experience two or more primary headache types that begin at different ages [25]. For this reason, in an attempt to test ID migraine in our population, we excluded patients diagnosed with TTH.

ID migraine tries to diagnose migraine based on the presence of photophobia, nausea, and pain- related disability. The questionnaire could also be adapted to pediatric age as these characteristics are frequently reported in children and adolescents with migraine. As our data also shows, the presence of these features provides ID migraine a valid tool despite the age of administration. In fact, in our sample in both age-related subgroups, ID migraine showed an excellent reliability profile despite the mean age of the two groups being different (8.58 years versus 14.75 years).

A previous study, conducted on patients under the age of 18 and suffering from migraine, found that most patients (83.4%) experienced at least one associated symptom between photophobia (79.2%) or nausea (64.5%) [40]. Another study showed that the best diagnostic items for migraine in children and adolescents are: 1) moderate or severe intensity, 2) pain aggravation by physical activity, and 3) pulsating quality of pain. Conversely, absence of photophobia or nausea, or no aggravation by physical activity were the most significant items in favor of TTH diagnosis [43]. In our sample photophobia was described by more than 60% of migraineurs, with a high specificity and PPV (81% and 94%, respectively). Nausea and vomiting were reported by 63% of migraine patients, while disability by 89%, thus supporting high reliability of ID Migraine (specificity 88% and 76%, PPV 95% and 94%). The presence of photophobia and phonophobia is less frequently described in pre-school children [25]. This could represent a problem for a possible ID migraine application to younger children. In the present study 73% of migraine patients reported photophobia, but children under 6 years were excluded from our observation.

Two previous studies investigated the validity of ID Migraine in pediatric age and their results disagreed (Table 3). Jin et al. achieved a specificity of 46.63% and a sensitivity of 39.71% investigated students aged from 7 to 15 years in a school context. The study was conducted in four primary schools where subjects were asked to complete a four-step questionnaire. In the first step, demographic features were recorded, and the presence of headache was screened. In the second one, 54 items were used to characterize the type of headache in migraine, TTH, cluster headache, and other headaches. In the third step, headache disability was recorded, while the fourth step was ID Migraine. The diagnosis obtained was migraine in 44.85% of subjects, TTH in 29.18%, cluster headache in 6.22%, and other headaches in 19.74% [23]. Zarigoflu et al. obtained a specificity of 71.1%, and a sensitivity of 62.1% from a three-steps interview administered to 12–17 years adolescent students. In the first phase, they were asked to fill a questionnaire concerning presence of headache and sociodemographic features. The second phase consisted in a face-to-face interview with students who had experienced headache. Lastly, the third step was the ID Migraine administration [27]. There are two main reasons which could explain the difference between both studies and our present results. First, our decision not to include TTH may have improved the sensitivity and specificity of the test. Indeed, in our control group patients suffered from headaches with characteristics clearly distinguishable from migraine and TTH. Second, we administered the test in a third-level headache center, which limits the generalizability of our results. For this reason, it is desirable to extend the administration to more settings. The generalizability of the tool provides its applicability to be stronger, allowing for earlier diagnosis.

**Table 3:** Studies investigated the validity of ID Migraine in pediatric age

	Sensitivity (%)	Specificity (%)	NPV (%)	PPV (%)
Jin et al	39.71	46.63	-	-
Zarigoflu et al	62.17	71.1	79.2	51.5

Specificity, sensitivity and negative (NPV) and positive (PPV) predictive values in previous validated ID migraine for pediatric age

The early diagnosis of migraine in pediatric age is crucial for several reasons: 1) it helps to manage symptoms effectively, 2) it improves child’s quality of life, and 3) it prevents potential long-term complications. The timely introduction of appropriate treatments is possible only after an early diagnosis. Effective management can reduce the frequency and intensity of the headache attacks, preventing them from disrupting the child’s daily life. Whether migraine is not properly diagnosed and treated, it can become more severe over time, thus leading to an increased risk of chronification and medication overuse. In addition, early diagnosis provides the opportunity for both children and their families to learn about migraine triggers, symptoms, and coping mechanisms [28, 29].

## Limitations

The main limitation of the present study is represented by a discrepancy between the number of migraine and non-migraine patients. This led to an underestimation of the NPV in all features and of the sensitivity in identifying nausea and photophobia. The selection of all subjects within a pediatric headache center led to the low number of control subjects. In order to improve the test's validation, it would be advantageous to have it distributed to a larger group of subjects from multiple headache centers. However, this work was based on the design and number of subjects used in previous migration ID validation work.

## Conclusion

Diagnosing pediatric migraine is a challenge because of the variable and evolving nature of symptoms, difficulties in children's communication, and the overlap with other conditions. To avoid complications or incorrect treatment, migraine should be hopefully diagnosed early in children and adolescents. Our results suggest that ID migraine can represent a valid tool for an early migraine diagnosis in both children over 6 years and adolescents.

## Data Availability

Source data of the study are available from the corresponding author on reasonable request.
